# Host resistance does not explain variation in incidence of male-killing bacteria in *Drosophila bifasciata*

**DOI:** 10.1186/1471-2148-4-52

**Published:** 2004-11-30

**Authors:** Zoe Veneti, Masanori J Toda, Gregory DD Hurst

**Affiliations:** 1 Department of Biology, University College London, 4 Stephenson Way, London NW1 2HE, UK; 2 Institute of Low Temperature Science, Hokkaido University, N19 W8, Kita-ku, Sapporo 060-0819, Japan

## Abstract

**Background:**

Selfish genetic elements that distort the sex ratio are found widely. Notwithstanding the number of records of sex ratio distorters, their incidence is poorly understood. Two factors can prevent a sex ratio distorter from invading: inability of the sex ratio distorter to function (failure of mechanism or transmission), and lack of drive if they do function (inappropriate ecology for invasion). There has been no test to date on factors causing variation in the incidence of sex ratio distorting cytoplasmic bacteria. We therefore examined whether absence of the male-killing *Wolbachia *infection in *D. bifasciata *in Hokkaido island of Japan, in contrast to the presence of infection on the proximal island of Honshu, was associated with failure of the infection to function properly on the Hokkaido genetic background.

**Results:**

The male-killer both transmitted and functioned well following introgression to each of 24 independent isofemale inbred lines carrying Hokkaido genetic backgrounds. This was maintained even under stringent conditions of temperature. We therefore reject the hypothesis that absence of infection is due to its inability to kill males and transmit on the Hokkaido genetic background. Further trap data indicates that *D. bifasciata *may occur at different densities in Hokkaido and Honshu populations, giving some credence to the idea that ecological differentiation could be important.

**Conclusions:**

The absence of the infection from the Hokkaido population is not caused by failure of the male-killer to function on the Hokkaido genetic background.

## Background

Selfish genetic elements that distort the sex ratio of their host are known widely in arthropods [1]. Despite over 70 years of research, we still do not fully understand the factors that dictate their presence or absence in different species in the field, nor the correlated question as to the factors causing variation in their frequency over geographical space within a species. A good approach to this problem is to examine the causes of variation within species, and in particular to identify the factors contributing to absence of elements from some populations in species known to bear the element in other areas.

Factors causing variation in prevalence/incidence over space may be either ecological or associated with differences in host genetic constitution. For instance, in the case of X chromosome meiotic drive in *Drosophila pseudoobscura*, frequency declines at both high latitude and high altitude. This is not associated with resistance to the action of the driver (no resistance is known), and variation in rates of multiple mating is suspected as a cause [2]. In contrast, in the case of X chromosome drive in *D. subobscura*, X drive is present in North Africa and absent in Europe. Here, absence of X drive is associated with the less efficient function of X drive on the European genetic background [3].

This study pertains to the factors affecting the incidence of male-killing bacteria. These bacteria pass from a female to her progeny, and kill any males they enter. Male-killers are common in insects, but an appreciation of the factors underlying their incidence is lacking [4]. In the first place, host genetic factors may affect the ability of a male-killer to transmit or function. Within *Drosophila prosaltans*, for instance, there is intra-population host genetic variation in refractoriness to male-killer action/transmission [5]. Thus, it is logical to conjecture that a male-killer can be absent from a population because the host has evolved resistance to its action or transmission. In the second place, presence/absence of infection can be determined by ecological, environmental or genetic variation that influences the benefit of male-killing to the bacterium [6]. For instance, laboratory studies by Jaenike *et al. *[7] have demonstrated that the number of females ovipositing within a patch is a key determinant of invasion success.

In this paper, we examine the factors that could cause incidence variation for the male-killing *Wolbachia *in *Drosophila bifasciata *in Japan. *Drosophila bifasciata *feeds on sap fluxes in deciduous forests, and sampling across 10 populations within Honshu island in Japan revealed a relatively constant frequency of infection, with between 5 and 7% of females infected with the male-killer [8]. The male-killer is a strain of *Wolbachia *still present on Honshu to this day [9].

In contrast to collections from Honshu, past surveys across Hokkaido, the North island of Japan, indicated flies in this area are not infected with the male-killer, despite the relative proximity of the sites to the infected populations in the Northern most sites in Honshu. In total, 559 flies from six locations within Hokkaido were tested, with no evidence of sex ratio distortion in any case [8]. We can be almost certain that the absence of infection is not due to the infection never arriving on this island. First, infection otherwise has a worldwide distribution, being found in Italian *D. bifasciata *populations [10]. Second, the 5 km wide Tsugara Straits between Honshu and Hokkaido may limit gene flow (and hence support differentiation), but are very unlikely to have been an absolute bar to the arrival of the infection.

We tested whether the absence of infection in Hokkaido was associated with an effect of host genotype on the efficiency of male-killer transmission or strength of male-killing ability. Beyond this, we examined whether trap collection data were consistent with difference in *D. bifasciata *ecology between Hokkaido and Honshu islands. Our results indicated that the Hokkaido genetic background supported the transmission and action of the male-killer even under stringent conditions, ruling out genetic differentiation as a cause of the absence of the male-killer from Hokkaido. We did observe higher capture rates of *D. bifasciata *in the island of Hokkaido, and future work should therefore be focussed on the degree to which ecological differences affecting the drive of the infection dictates incidence in this species.

## Results

### Sex ratio of Hokkaido flies

Twenty-eight female flies were collected from the field in Hokkaido. Of these, 4 failed to produce progeny. The remaining 24 all produced a normal sex ratio, consistent with continued absence of the male-killing trait in Hokkaido.

### Intensity of male-killing on the Hokkaido genetic background

We tested whether the Hokkaido genetic background supported the male-killing *Wolbachia *from Honshu by introgression of the infection onto the Hokkaido genetic background. The progeny from the 24 Hokkaido females were maintained as isofemale inbred lines for three generations to capture genetic variation within them. The male-killing *Wolbachia *from Honshu was then placed onto each inbred background via crossing infected females from Honshu to males from each of the Hokkaido lines, with subsequent generations being maintained through further crossing to males from the appropriate Hokkaido line.

The sex ratio produced following introgression of the infection to the Hokkaido genetic background was female biased and penetrance of the male-killing phenotype was perfect in the first generation. A few males appeared sporadically in six of the 24 lines in one or more subsequent generations, with highest frequency in lines 15 and 25 (Table 1). However, no males were produced in the F4 in any case, indicating no loss of infection or repeatable resistance to male-killer action. We particularly maintained observation over lines 15 and 25 over four subsequent generations, and adult males were not observed in the culture over this period (data not shown). These data are broadly comparable with data from Honshu control lines, where 4 of 27 lines showed sporadic male production.

**Table 1:** The sex ratio produced during introgression of the male-killing infection to the Hokkaido genetic background. 'All-female' classification represent cases where both replicates produced all female broods. Where males were produced within a female-biased sex ratio, data is given separately for each replicate of the isofemale line.

Generation	Sex ratio	No. of lines	Line-replicate: male
n			progeny/total
F1	All female	24	
	Female biased	0	
F2	All female	21	
	Female biased	3	15-1: no males. 15-2: 2/74
			21-1: 1/32. 21-2: no males
			25-1 no males. 25-2: 1/10
F3	All female	19	
	Female biased	5	12-1 no males. 12-2: 1/18
			15-1: 2/27 15-2: 2/52
			17-1: 2/20 17-2: 1/46
			20-1: 1/10 20-2: no males
			25-1: 1/40 25-2: no males
F4	All female	24	
	Female biased	0	

### Effect of stringent temperatures

The lines above were moved to 23.5°C, the upper temperate before thermal induced loss of infection occurs in Honshu flies [11], and we maintained the lines at this temperature by backcrossing to males from the source uninfected Hokkaido line as before, for four further generations. No effect of elevated temperature on the penetrance or transmission of the male-killing trait was observed on the Hokkaido genetic background (Table 2). Sporadic males were observed in 4 of 20 lines over the four generations. However, males were never observed in both replicates within a line, nor were they ever observed in more than one generation within a line. Notably, none were produced in the final generation, indicating the infection was perfectly transmitted during the experiment. Control lines from Honshu maintained production of all female broods, in agreement with past observations.

**Table 2:** The sex ratio in male-killer infected isofemale lines from Hokkaido following transfer of the introgressed infected lines to 23.5°. 'All-female' classification represents cases where both replicates produced all female broods. Where males were produced within a female-biased sex ratio, data is given separately for each replicate of the isofemale line.

Generation	Sex ratio	No. of lines	Line-replicate: male
n			progeny/total
F1	All female	20	
	Female biased	0	
F2	All female	20	
	Female biased	0	
F3	All female	16	
	Female biased	4	11-1 no males 11-2: 1/21
			19-1: 1/28 19-2: no males
			23-1: 1/37 23-2: no males
			25-1: 1/11 25-1: no males
F4	All female	20	
	Female biased	0	

### Collection rates of *D. bifasciata *in traps

We captured flies in the field and scored the samples for both absolute capture rate of *D. bifasciata*, and capture rate relative to other species of *Drosophila*. The capture rate of *D. bifasciata *was substantially higher in all four samples taken in Hokkaido province (Misumai, Koryukozan, Tomakomai, Matsumae) than in the two samples from the Northern Honshu populations (Mimmaya, Morioka) and the population from Mid Honshu (Kiyosumi). Increased capture rate in Hokkaido was also reflected in an increase in the proportion of all drosophilids sampled that were *bifasciata *(Table 3). This is consistent with the idea that the ecology of *D. bifasciata *varies between Honshu and Hokkaido, and that this may cause the presence of the infection in one island and absence in the other.

**Table 3:** Catch rate of *D. bifasciata *in seven locations within Japan during early-mid October between 1973 and 1984. Catch rate is given as mean per trap per day, with number of traps and number of days trapped in parentheses. Proportion of catch that was *bifasciata *is given across all traps and days, with total *Drosophila *catch in parentheses.

Island	Location		*bifasciata *caught per day/ per trap (traps, days)	*bifasciata *as proportion of catch (n)
Hokkaido	Misumai	42°57' N 141°16' E	4.67 (5, 14)	13.6% (2407)
	Koryukozan	42°51' N 141°17' E	8.38 (5, 17)	15.94% (4458)
	Tomakomai	42°43' N 141°36' E	6.48 (6, 14)	2.01% (27033)
	Matsumae	41°26' N 140°08' E	2.54 (8, 7)	2.48% (5726)
Honshu	Mimmaya	41°10' N 140°24' E	0.21 (8, 7)	0.51% (2342)
	Morioka	39°15' N 141°10' E	0.79 (4, 7)	0.82% (2683)
	Kiyosumi	35°10' N 140°10' E	0.00 (4, 7)	0 (903)

## Discussion

Previous study has shown that male-killing *Wolbachia *are absent from *D. bifasciata *in Hokkaido province of Japan, despite the infection being present in neighbouring Honshu. In this study, we have demonstrated that the absence of male-killing *Wolbachia *in the Hokkaido population is not caused by the inability of the male-killing *Wolbachia *to operate on the Hokkaido host genetic background. In contrast, the male-killer was perfectly transmitted in all 24 lines tested (after 4 generations of introgression, no males were produced in any of the lines). This high efficiency was maintained even at the threshold for complete male-killing in Honshu, 23.5 C (after a further 4 generations, all 20 lines still had all female broods). Thus, the male-killer is proficient at being transmitted and killing males on the Hokkaido background even under relatively stressful environmental conditions. Further, we continue to maintain the infection on the Hokkaido background (we are now at generation 15) without any loss of the infection and without appearance of males. Thus, it is not tenable to argue that the hosts themselves are not genetically suitable for the function of male-killer, at least on an ecological timescale. This situation contrasts with the case of meiotic drive in the related fly *D. subobscura*, where absence of drive in Europe was associated with refractoriness to the action of the sex ratio distorting element.

In the absence of variation in the ability of the male-killer to function on the different genetic backgrounds, the question arises as to the features that cause infection to be absent from the Hokkaido population. Our study did reveal differences in trap collection rates between the populations of Hokkaido and those of Northern Honshu, with *bifasciata *captured at lower rate in the populations from Honshu than in Hokkaido. Thus, ecological heterogeneity is a possible source of the incidence pattern. There are three parameters in male-killer dynamics that may be environmentally influenced. First, the advantage to male-killing may not be as strong in Hokkaido populations. Second, the cost of infection to female flies may be higher in Hokkaido than in Honshu. Third, the transmission efficiency may alter, mediated via elevated temperature, or possibly by reduced overwinter temperature.

In our view, the latter factor is unlikely to be driving the observed pattern. It is notable that the survey of Ikeda revealed the infection to be present in Northern Honshu, but not in Southern Hokkaido. Given these two areas are geographically and climatically very close, temperature differences have poor explanatory power. Explanations based on temperature are also weak because this species exhibits a degree of homeostasis in temperature, moving to elevated altitudes to avoid excess temperature.

This leaves us with two hypotheses to explain absence of infection in Hokkaido. The first is that there is a weaker advantage to male-killing in Hokkaido than on Honshu, such that there is insufficient drive to maintain the bacterium or permit its spread. The advantage of male-killing to the bacterium depends upon the number of females ovipositing in a single patch [7]. If the high density of *bifasciata *observed in Hokkaido translates into many females laying eggs in a single sap flux, male death will not greatly increase the survival of infected females over uninfected, and infection will decline in frequency. The second factor that may cause infection to decline in frequency is if costs of infection are higher in Hokkaido than in Honshu. This factor can be ecologically contingent. Ikeda demonstrated that the relative fitness of infected flies compared to uninfected flies was lower under high densities in the laboratory [8]. Thus, if the high density of the adult fly we observed corresponds to a high density of larvae within a single sap flux, the direct cost of infection would be higher, and the infection would be expected to be less common or absent.

Aside from these possibilities, which we consider most likely, other ecological discontinuities between Hokkaido and Honshu deserve investigation. Some symbionts, for instance, give the host protection against parasitoids [12], and thus differences in parasitoid infection rates could affect the frequency of a symbiont. Co-existing heterospecific competitors may also diminish the benefit of male-killing; if there are many species ovipositing within a patch, the advantage to male-killing may decline. Thus, the intensity of inter-specific competition also deserves investigation. Finally, the existence of other 'competing' inherited microorganisms should be excluded as a reason for the absence of the male-killing *Wolbachia *from Hokkaido.

## Conclusions

It is not variation in the ability of *Wolbachia *to function on different host genetic backgrounds that drives presence or absence of infection in the *D. bifasciata*- male-killing *Wolbachia *system. We have demonstrated that despite being absent from Hokkaido, *Wolbachia *can both be maintained and express male-killing on the Hokkaido host genetic background. We observe that the two populations show differences in trap capture rates, and argue that either ecologically contingent benefits or ecologically contingent costs of infection may explain presence and absence of infection in this species, and that future research be focussed at this level.

## Methods

### Source of wild flies for introgression

Twenty eight wild female *D. bifasciata *were collected on the campus of Hokkaido University, Sapporo (43°4'56"N, 141°20'21"E), Japan, in May 2003. These were then brought into the laboratory, where they were maintained individually in vials at 21°C on a modified cornmeal-agar diet (70 g sucrose, 60 g maize meal, 15 g yeast extract, 10 g agar, 2.5 g nipagin in a total volume of 1 liter). These female were checked for the presence of the male-killing trait through scoring of the sex ratio, and maintained by sib-sib inbreeding (2 males, 2 females) for three generations to make inbred isofemale lines. In total, 24 isofemale inbred lines were created (4 lines went extinct), which were maintained thenceforth by simple tossing every three weeks.

### Introgression of the infection onto the Hokkaido genetic background

The genetic background of each of the 24 uninfected Hokkaido lines was then independently crossed onto the male-killer infected cytotype over four generations. To this end, 4 males were taken from each of the 24 Hokkaido lines, and mated to 4 *Wolbachia *infected virgin females extracted from a culture derived from Honshu island, Japan (established in [9]). This procedure was performed twice for each Hokkaido inbred line to give 24 introgression lines, each replicated with two replicates. Following this initial cross, introgression of the appropriate Hokkaido nuclear background continued for four generations, on each occasion four female offspring of each line being backcrossed to males from the respective Hokkaido uninfected inbred line. Flies were kept at 21°C throughout the experiment, and the sex of each line scored at each generation (n>10 individuals in every case). As a control against spontaneous loss of infection not associated with genetic differentiation, the male-killer was concurrently maintained on the Honshu genetic background, in lines maintained by backcrossing to individual isofemale lines (as established in [11]) with likewise monitoring of sex ratio.

### Temperature effect

Temperature is known to affect the stability of the male-killing trait, and previous study demonstrated that an upper threshold of 23.5 C existed for stable maintenance of the infection on the Honshu genetic background [11]. We tested whether the infection remained stable at this stringent temperature on the Hokkaido genetic background. To this end, 20 of the above introgressed fly lines were transferred from 21° to the 23.5°, and the sex ratio of the offspring recorded for four further generations, with the lines maintained by backcrossing to males from the appropriater parental Hokkaido uninfected line as before. As a control, four Honshu isofemale lines were concurrently maintained at 23.5°.

### Collection rates of *D. bifasciata *in traps

**Figure 1:**
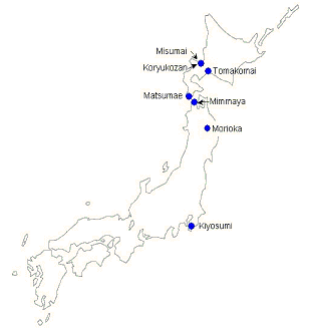
Collection sites for *D. bifasciata *in Japan

Evidence of differences in fly density can be derived from sampling the *Drosophila *communities. *Drosophila *communities were sampled in 4 deciduous forests in Southern Hokkaido, 2 sites in Northern Honshu, and one site in Mid Honshu, in early-mid October over a number of years between 1973 and 1984 (Figure 1). Collections were carried out using traps baited with fermented banana suspended from the canopy during early-mid October. The traps were especially designed for retaining trapped insects in a bottle of fixative solution and set vertically at different heights from the floor [13,14]. Since *D. bifasciata *is a typical forest-canopy dweller, sampling from the forest canopy is essential for estimating its population density. These traps were cleared 7 or 10 days after setting, and the capture rate of *bifasciata *per trap per day calculated at each locality to represent the density of this fly in this region. All drosophilid flies were identified, and the proportion of flies caught that were *bifasciata *recorded.

## Authors' contributions

ZV helped with the design of the crossing scheme, conducted the crosses involved, and performed the analysis of these crosses. MT designed and conducted the field sampling and scored trap collections. GH conceived the project, organised collection, helped with design of the crossing scheme, and wrote the paper. All authors read and commented on drafts of the manuscript, and approved the final manuscript.
